# Multiple interval QTL mapping and searching for *PSTOL1* homologs associated with root morphology, biomass accumulation and phosphorus content in maize seedlings under low-P

**DOI:** 10.1186/s12870-015-0561-y

**Published:** 2015-07-07

**Authors:** Gabriel C Azevedo, Adriana Cheavegatti-Gianotto, Bárbara F Negri, Bárbara Hufnagel, Luciano da Costa e Silva, Jurandir V Magalhaes, Antonio Augusto F Garcia, Ubiraci GP Lana, Sylvia M de Sousa, Claudia T Guimaraes

**Affiliations:** Departamento de Biologia Geral, Universidade Federal de Minas Gerais, Avenida Presidente Antônio Carlos, 6627, Belo Horizonte, MG 31270-901 Brazil; Departamento de Genética, Escola Superior de Agricultura Luiz de Queiroz, Universidade de São Paulo, Caixa Postal 83, Piracicaba, SP 13400-970 Brazil; Departamento de Bioengenharia, Universidade Federal de São João del-Rei, Praça Dom Helvécio, 74, São João del-Rei, MG 36301-160 Brazil; Núcleo de Biologia Aplicada, Embrapa Milho e Sorgo, Rodovia MG 424, km 65, Caixa Postal 151, Sete Lagoas, MG 35701-970 Brazil

**Keywords:** Phosphorus acquisition, Protein kinase, SNP marker, OsPSTOL1, *Zea mays*

## Abstract

**Background:**

Modifications in root morphology are important strategies to maximize soil exploitation under phosphorus starvation in plants. Here, we used two multiple interval models to map QTLs related to root traits, biomass accumulation and P content in a maize RIL population cultivated in nutrient solution. In addition, we searched for putative maize homologs to *PSTOL1*, a gene responsible to enhance early root growth, P uptake and grain yield in rice and sorghum.

**Results:**

Based on path analysis, root surface area was the root morphology component that most strongly contributed to total dry weight and to P content in maize seedling under low-P availability. Multiple interval mapping models for single (MIM) and multiple traits (MT-MIM) were combined and revealed 13 genomic regions significantly associated with the target traits in a complementary way. The phenotypic variances explained by all QTLs and their epistatic interactions using MT-MIM (23.4 to 35.5 %) were higher than in previous studies, and presented superior statistical power. Some of these QTLs were coincident with QTLs for root morphology traits and grain yield previously mapped, whereas others harbored *ZmPSTOL* candidate genes, which shared more than 55 % of amino acid sequence identity and a conserved serine/threonine kinase domain with *OsPSTOL1*. Additionally, four *ZmPSTOL* candidate genes co-localized with QTLs for root morphology, biomass accumulation and/or P content were preferentially expressed in roots of the parental lines that contributed the alleles enhancing the respective phenotypes.

**Conclusions:**

QTL mapping strategies adopted in this study revealed complementary results for single and multiple traits with high accuracy. Some QTLs, mainly the ones that were also associated with yield performance in other studies, can be good targets for marker-assisted selection to improve P-use efficiency in maize. Based on the co-localization with QTLs, the protein domain conservation and the coincidence of gene expression, we selected novel maize genes as putative homologs to *PSTOL1* that will require further validation studies.

**Electronic supplementary material:**

The online version of this article (doi:10.1186/s12870-015-0561-y) contains supplementary material, which is available to authorized users.

## Background

The increasing demand for agricultural production poses a global challenge to improve the phosphorus (P) use efficiency in plants due to its low availability in a large proportion of arable lands [1, 2]. Plants uptake phosphorus from the soil in the orthophosphate forms (P_i_), which are available at low concentration in the soil solution [3]. In a large fraction of soils, P is tightly fixed to the clay’s surface, which requires high amounts of phosphate fertilizers for high-yielding farming systems, increasing production costs and hampering soil fertility management [3–5]. However, low-input farmers have limited access to phosphate fertilizer, which is the second most used fertilizer for plant growth [6]. Maize is the most common grain produced worldwide and a major staple food in Africa and Latin America [7], where soils often show limited P availability. Thus, improving maize P-use efficiency is expected to increase yield stability and, consequently, food security [1, 4, 8].

Plants have evolved two major strategies to overcome P limitation in the soil, which are P internal utilization and P uptake [3]. P internal utilization mechanisms involve transport, partitioning and remobilization of P within the plant, whereas the mechanisms that increase P uptake are associated with alterations in the root system, interactions with microorganisms, and chemical modifications of the rhizosphere [3]. Indeed, P acquisition efficiency has been considered from two to three times more important than P internal utilization to explain the variability for P-use efficiency in tropical maize genotypes evaluated in low- and high-P soils [9]. Considering the limited mobility and low P concentration in the soil, mechanisms related to P acquisition are greatly dependent of the proximity of this nutrient to the root system [3, 10]. Thus, a well-developed root system should be an important adaptation mechanism to maximize soil exploitation, enabling plants to improve P acquisition efficiency [11–13]. Studies have shown that plants that are more efficient in P acquisition presented higher root:shoot dry weight ratios [14, 15], reduced root diameters [16], longer and denser root hairs [17], increased lateral roots [18], greater lateral branching and shallower basal roots [17, 19]. These changes in root morphology are key strategies used by plants to improve soil exploitation at a minimal metabolic cost [5, 20].

Root morphology is controlled by multiple genes in maize [13, 21], but only a few of them such as *roothairless* (*Rth1*) [22], *brittle stalk-2-like protein 3* (*Bk2l3*) [23], and *rootless concerning crown and seminal roots* (*Rtcs*) [24] have been cloned and characterized. However, an appropriate strategy to dissect these traits is through quantitative trait loci (QTL) mapping. Indeed, several QTLs were mapped for root traits under contrasting conditions of P availability in nutrient solution [25–27], in glasshouse [28] and in the field [29–32]. These QTLs individually explained from 1 to 14 % of the phenotypic variation, confirming the genetic complexity of root traits. These studies were carried out using composite interval mapping (CIM) strategy, however, multiple interval mapping (MIM) [33] offers a significant improvement on both statistical power and precision for detecting main and epistatic QTLs over CIM. This happens because MIM utilizes the estimated positions of QTLs as cofactors in the multiple regression model, whereas CIM utilizes the nearest markers to the estimated QTL as cofactors [34]. Besides all statistical advantages achieved by MIM, it still cannot capture the genetic correlation that might exist between traits. The multiple-trait multiple interval mapping (MT-MIM) method [35], which is an extension of MIM, applies multiple regression on a multiple dimensional (traits) space context, which enables it to capture information that might be available from the existing genetic correlation between traits, therefore, boosting the precision and power to detect QTLs [35]. To the best of our knowledge, this method has never been applied to map QTLs with effects on root morphology traits in maize.

In rice, a major QTL controlling phosphorus uptake (*Pup1*) was mapped to chromosome 12, explaining approximately 80 % of the phenotypic variance of this trait [36]. Rice near isogenic lines (NILs) carrying the *Pup1* QTL showed a three-fold increase in P uptake and enhanced root surface area when grown in P-deficient soil [36, 37]. Additionally, irrigated and upland rice varieties introgressed with *Pup1* showed a significant improvement in grain yield in different low-P soils compared to their parents [38, 39]. The gene underlying the *Pup1* locus was identified and named *Phosphorus-starvation tolerance 1* (*PSTOL1*), which encodes a serine/threonine kinase of the LRK10L-2 subfamily [40]. The overexpression of *PSTOL1* in two transgenic rice varieties enhanced the grain yield over 60 % under low-P conditions due to larger root system (i.e., root length, and total root surface area), which also improved the uptake of P and other nutrients [40]. Furthermore, Hufnagel *et al*. [41] showed that sorghum homologs to *OsPSTOL1* were associated with enhanced P uptake and grain yield in sorghum grown in a low-P soil due modifications on root system morphology and architecture. A remarkable conservation of protein-encoding genes among maize, sorghum and rice has been confirmed *in silico* based on genome sequencing comparison, once approximately 89 % of the 11,892 maize gene families predicted in the B73 genome were shared with rice and sorghum [42]. Of these, genes encoding important adaptive traits are expected to be conserved among these grass species.

In order to better understand the genetic basis of root morphology and P acquisition related traits, as well as the relationship between these traits, a path analysis and a QTL mapping study were carried out in a maize RILs in nutrient solution cultivated under low P. We also integrated the QTL mapping, sequence comparison and expression analysis to identify putative homologs to *PSTOL1* in maize.

## Results

### Transgressive segregation of phenotypic traits in RILs

**Fig. 1:**
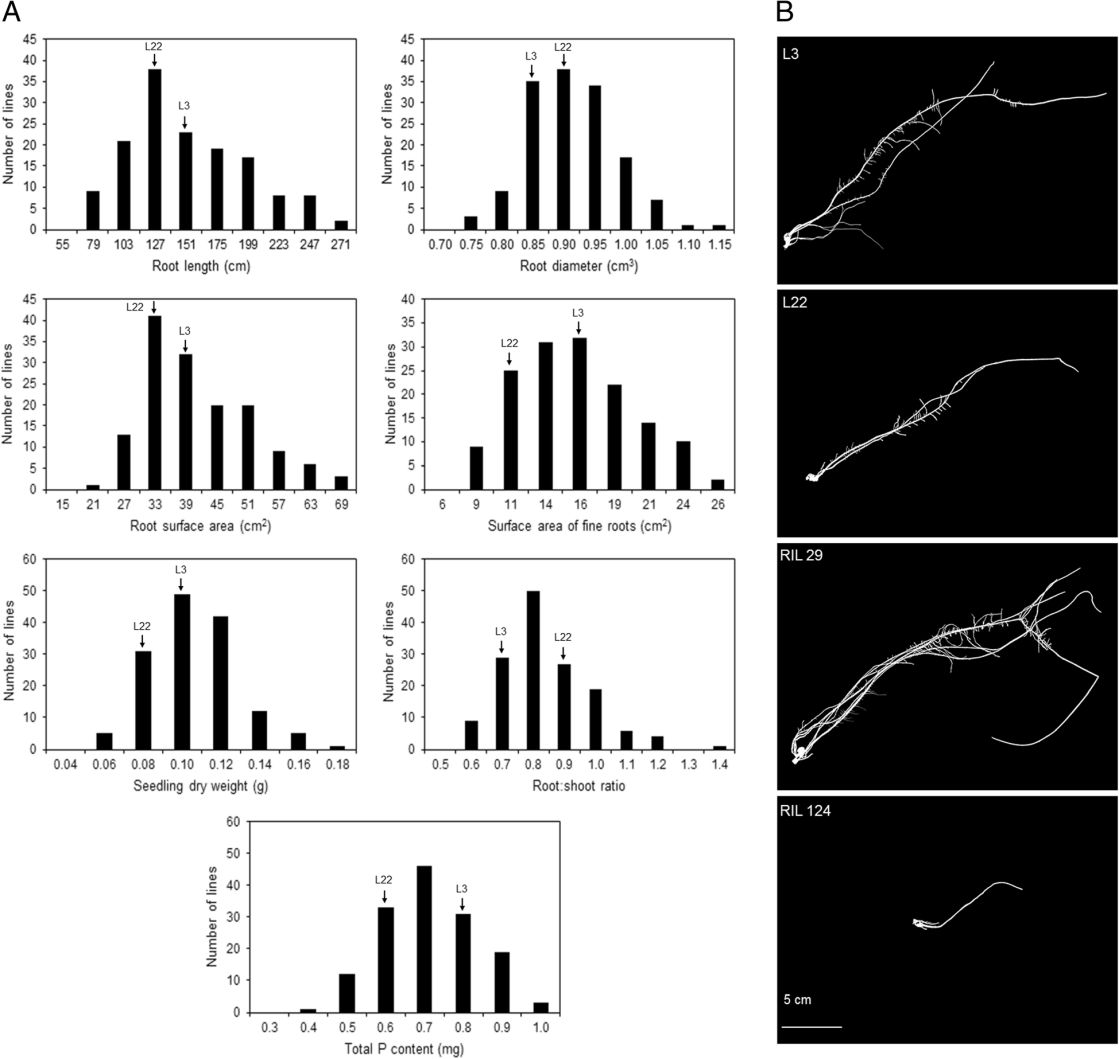
**a** Frequency distribution of traits evaluated in 145 maize RILs grown in low-P (2.5 μM). The P-efficient (L3) and the P-inefficient (L22) parental lines are indicated by arrows. **b** Root system of the parental lines (L3 and L22) and two extreme RILs (RIL 66 and 113) after 13 days cultivated in nutrient solution under low-P

Significant genetic variation in root morphology traits, biomass accumulation and phosphorus content in the seedlings were observed for the RIL population with high broad sense heritability estimates, which ranged from 0.65 for root:shoot dry weight ratio to 0.82 for root length (Additional file 1: Table S1). The P-efficient line, L3, tended to present superior phenotypic measurements for all traits compared with the P-inefficient line L22, with the exception of root diameter and root:shoot ratio (Fig. 1a). The range of the phenotypic variation in the RILs was larger than both parents, suggesting transgressive segregation for all phenotypic traits (Fig. 1a). The RILs showing extreme root systems in comparison with their parental lines were highlighted in the Fig. 1b. The parental lines belonged to distinct heterotic groups (L3, flint and L22, dent) [43], were genetically divergent based on SNP markers [44] and contrasted for grain yield in low-P soil [45] and for root morphology traits in nutrient solution [46].

### Surface area is an important root trait contributing to seedling dry weight and P content

Root length, root surface area and surface area of fine roots were high and positively correlated with each other (Table 1). The correlation coefficients among these traits exhibited comparable magnitudes to those observed in a sorghum diversity panel composed of 287 accessions [41] and in a group of 30 maize lines [46]. These root traits also showed strong correlation coefficients with total seedling dry weight (0.77 to 0.86), moderate correlations with total P content in the seedling (0.31 to 0.48), and negative correlations with root:shoot ratio (−0.29 to −0.38) (Table 1). In contrast, root diameter was negatively correlated with root length, root surface area and total seedling dry weight, but no significant correlation was found with total P content. The negative correlation between root length and root diameter (−0.62) was similar to the coefficients obtained for root diameter with lateral (−0.65) and non-lateral (−0.68) roots in temperate maize RILs [18].

**Table 1:** Phenotypic correlation coefficients (*r*) among traits evaluated in the RILs under low-P condition in nutrient solution

Traits	RL	SA2	RD	R:S	TDW	Pcont
SA	0.98**	0.80**	−0.48**	−0.38**	0.86**	0.39**
RL		0.69**	−0.62**	−0.38**	0.79**	0.31**
SA2			0.03	−0.29**	0.77**	0.48**
RD				0.26**	−0.25**	0.14
R:S					−0.43**	−0.02
TDW						0.58**

Traits: root surface area (SA), root length (RL), surface area of fine roots (SA2), root diameter (RD), root:shoot dry weight ratio (R:S), total seedling dry weight (TDW), and total P content (Pcont)

Correlation coefficients followed by ** are significant at *p* < 0.01

To further investigate the relative importance of root traits on seedling dry weight and P content, we performed a path analysis, using the root traits as explanatory variables and the total seedling dry weight and P content as dependent variables. As P content was the product of total seedling dry weight and P concentration as previously proposed [30, 31], both dependent variables were significantly correlated (0.58) and were evaluated separately. The partitioning of the correlation coefficients revealed that root length had the lowest direct effect on total seedling dry weight (−4.831) and P content (−0.997), but had a strong indirect effect through root surface area (5.816 and 1.565, respectively) (Table 2). Thus, the negative direct effect of root length was counterbalanced by the indirect effect via root surface area, probably due to the high positive correlation between these traits (*r* = 0.98). A similar pattern was observed for surface area of fine roots, which was also positively correlated with root surface area (*r* = 0.8). This trait exerted a minor negative direct influence on total seedling dry weight (−0.681) and P content (−0.117), which were mitigated by the positive indirect effect via root surface area (4.748 and 1.277, respectively). Root diameter also played a more important effect indirectly via root surface area on both variables, masking its direct contribution, which corroborates with the negative correlation between these root traits (*r* = −0.48). Therefore, root surface area contributed the highest direct effect on total seedling dry weight (5.935) and P content (1.597) and mediated an important proportion of the indirect effects of the other root traits on the dependent variables.

**Table 2:** Path analysis showing the partitioning of the phenotypic correlations into direct and indirect effects of root traits on total seedling dry weight and P content

Independent variables		Dependent variables	
		Total dry weight	Total P content
Root surface area	Direct effect	5.935	1.597
	Indirect effect via root length	−4.734	−0.977
	Indirect effect via surface area of fine roots	−0.544	−0.094
	Indirect effect via root diameter	0.203	−0.135
	Phenotypic correlation (*r*)	0.86	0.39
Root length	Direct effect	−4.831	−0.997
	Indirect effect via surface area	5.816	1.565
	Indirect effect via surface area of fine roots	−0.462	−0.081
	Indirect effect via root diameter	0.267	−0.177
	Phenotypic correlation (*r*)	0.79	0.31
Surface area of fine roots	Direct effect	−0.681	−0.117
	Indirect effect via root length	−3.284	−0.678
	Indirect effect via surface area	4.748	1.277
	Indirect effect via root diameter	−0.012	0.008
	Phenotypic correlation (*r*)	0.77	0.48
Root diameter	Direct effect	−0.424	0.281
	Indirect effect via root length	3.043	0.628
	Indirect effect via surface area	−2.848	−0.766
	Indirect effect via surface area of fine roots	−0.020	−0.003
	Phenotypic correlation (*r*)	−0.25	0.14
	Coefficient of determination	0.869	0.295

Thus, the path analysis clarified the direct and indirect importance of a greater root surface area, which is a combination of longer roots with smaller diameters, to improve total dry weight and P content in the seedlings under P deficiency. This root morphology also promoted an additional advantage for shoot over root development, confirming that the investment in root growth was beneficial to P acquisition as discussed by Zhu and Lynch [18].

### Two distinct QTL mapping strategies reveal complementary results

**Fig. 2:**
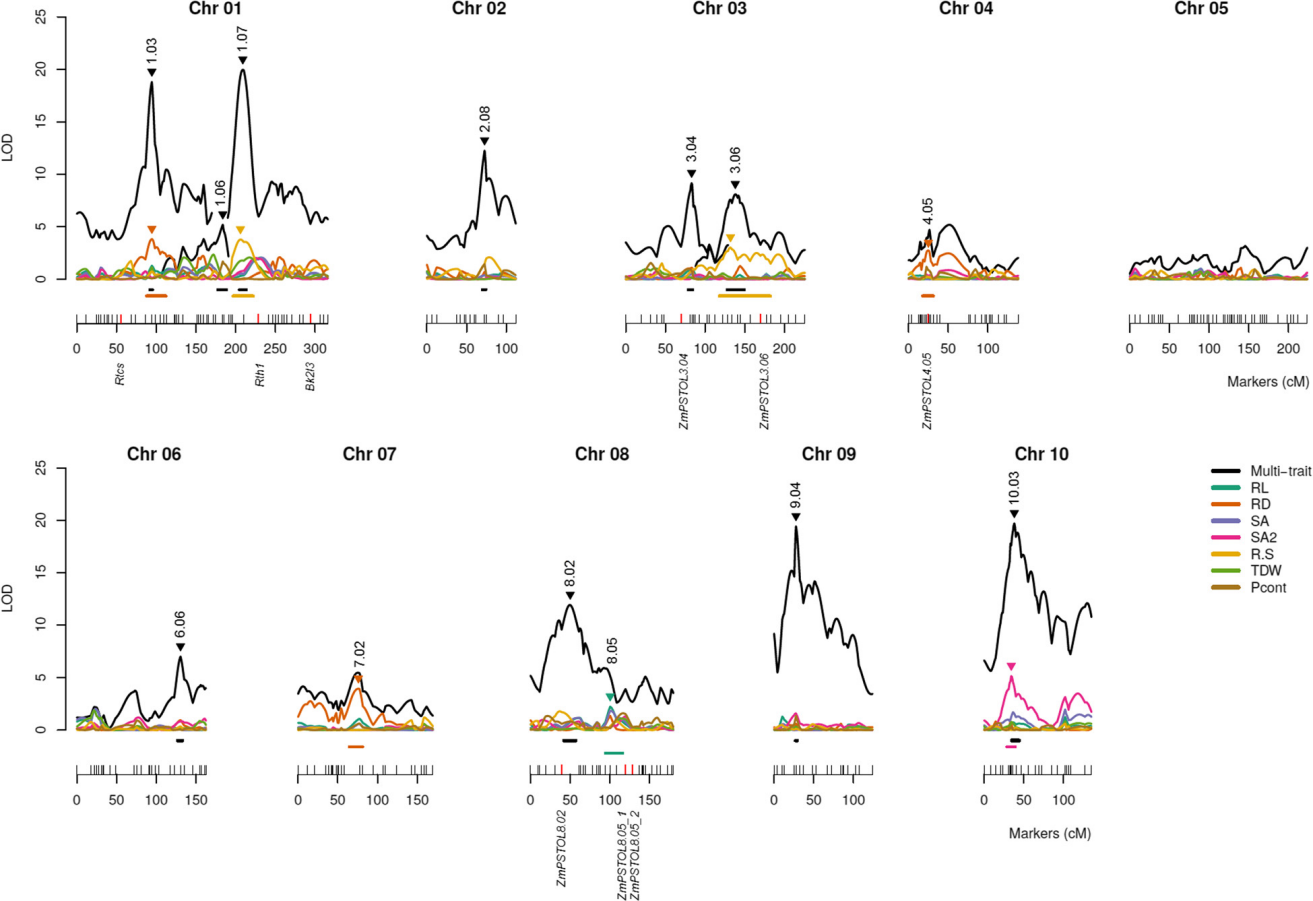
QTLs identified for root traits, seedling dry weight and P content using single and multiple traits MIM analyses. The markers are represented as vertical traces along the horizontal lines, which represent the chromosomes and are numbered in centiMorgans (cM). The candidate genes are depicted below the red vertical traces. QTL profiles for single trait MIM are shown as colored lines according to the legend for root length (RL), root average diameter (RD), root surface area (SA), surface area of fine roots (SA2), root:shoot ratio (R:S), total seedling dry weight (TDW) and total P content (Pcont). Multi trait QTL profiles are shown as black line. The QTL peaks are depicted with an inverted triangle colored according to the legend followed by the bin. The confidence interval (95 %) for each QTL is represented by a horizontal line above the chromosomes colored according to the legend

A linkage map was constructed using 292 markers that covered 1787.5 cM of the maize genome, with an average interval of 6.1 cM between adjacent markers (Additional file 2: Figure S1). In addition to SSR and SNP markers, six *ZmPSTOL* candidate genes and three genes previously associated with root morphology (*Rth1*, *Bk2l3,* and *Rtcs*) were mapped to their predicted physical positions. Multiple interval mapping models for single (MIM) and multiple traits (MT-MIM) provided statistical evidence for 13 genomic regions harboring QTLs on all maize chromosomes, with the exception of chromosome 5 (Tables 3 and 4). The QTL regions were named using the trait initials if they were detected through single trait analysis or as “multi” if they were detected by multiple trait analysis, followed by their genetic position in bin (Fig. 2). A bin is the interval of approximately 20 cM between two core markers previously defined and mapped in maize [47], which are designated with the chromosome number followed by a two-digit decimal.

**Table 3:** Quantitative trait loci (QTLs) identified using single trait-multiple interval mapping analysis for root length (RL), root diameter (RD), surface area of fine roots (SA2) and root:shoot ratio (R:S) under low-P

Trait	QTL^a^	Bin	cM^b^	Marker / Position (Mbp)	LOD	Flanking Markers^c^ / Position (Mbp)		R^2^ (%)^d^	Effect^e^	R^2^_T_ (%)^f^
RL	*qRL8.05*	8.05	100.4	PZA00766_1	2.24	PHM934_19	*ZmPSTOL8.05_1*	6.87	−0.271**	6.87
				133.8		116.8	152.0			
RD	*qRD1.03*	1.03	94.4	umc1073	3.80	bnlg1083	PZA03742_1	9.60	0.307***	25.64
				32.9		27.5	44.5			
	*qRD4.05*	4.05	25.0	*ZmPSTOL4.05*	2.74	PHM15427_11	PHM3587-6	6.84	0.270***	
				39.8		33.9	59.4			
	*qRD7.02*	7.02	76.0	PZA01690_7	3.95	PZA01933_3	PZA01946_7	10.01	−0.331***	
				123.1		98.1	123.6			
SA2	*qSA2_10.03*	10.03	34.2	PHM2770_19	5.16	PHM1155_14	PZA01877_2	15.12	−0.393***	15.12
				72.6		62.1	77.5			
R:S	*qRS1.07*	1.07	206.0	PHM12693_8	3.76	PZA01963_15	PZA03301_2	10.85	0.377***	16.47
				223.5		203.7	240.6			
	*qRS3.06*	3.06	132.0	PZA02212_1	3.00	PZA00186_4	PZA01154_1	8.53	0.310***	
				174.5		165.8	216.0			

^a^QTLs are named using the trait initials followed by their genomic position in bin

^b^cM and Mbp indicate the marker position in centiMorgans and in mega base pairs at maximum LOD value

^c^Flanking markers are based on −1.5 LOD support interval

^d^R^2^: Ratio of the genotypic variance of the QTL effect to the phenotypic variance, times 100

^e^Effects measured as standard deviation from the progeny mean: Positive values indicate that L3 carries the allele for an increase in the trait, and negative values indicate that L22 contributes the allele for an increase in the trait. Effect significance based on *p-values* estimated via score statistics resampling (^+^ *p* < 0.1, *p < 0.05, **p < 0.01, ***p < 0.001)

^f^R^2^_T_: genotypic variance of the full model

**Table 4:** Quantitative trait loci (QTLs) identified using multiple traits-multiple interval mapping analysis for root length (RL), root diameter (RD), root surface area (SA), surface area of fine roots (SA2), root:shoot ratio (R:S), total seedling dry weight (TDW) and total P content (Pcont)

QTL^a^	Bin	cM^b^	Marker/Position (Mbp)	LOD	Flanking markers^c^/Position (Mbp)		Main effect^d^ / R^2^ (%)^e^						
							RL	RD	SA	SA2	R:S	TDW	Pcont
*qMulti1.03*	1.03	94.4	umc1073	18.4	bnlg1083	PZA03742_1	−0.109	0.163*	−0.075	0.110	−0.168*	−0.147*	0.026
			32.9	9	27.5	44.5	1.18	2.64	0.56	1.19	2.82	2.14	0.65
*qMulti1.06*	1.06	183.	PZA00619_3	5.07	bnlg1598	umc1335	0.093	−0.028	−0.084	0.078	−0.054	−0.004	0.210**
		8	195.4		187.8	196.9	0.85	0.08	0.70	0.60	0.27	0.00	4.33
*qMulti1.07*	1.07	209.0	PHM114614_22	20.01	PZA01963_15	PHM12693_8	−0.185*	0.010	−0.231**	−0.224**	0.373***	−0.299***	−0.138^+^
			205.6		203.7	223.5	3.03	0.01	4.73	4.41	12.28	7.89	1.67
*qMulti2.08*	2.08	72.7	PZA01885_2	16.10	PZA02077_1	PZA01885_2	0.116	−0.109	0.083	−0.041	−0.111	−0.049	−0.262***
			206.9		206.5	206.9	1.28	1.13	0.65	0.02	1.16	0.23	6.50
*qMulti3.04*	3.04	83.0	PZA00297_2	7.90	*ZmPSTOL3.04*	PHM5502_31	0.217**	−0.216**	0.192*	0.119^+^	0.124^+^	−0.023	−0.059
			39.9		20.2	67.2	4.28	4.25	3.34	1.29	1.40	0.05	0.31
*qMulti3.06*	3.06	138.	PZA01962	8.25	PZA02212_1	PZA03735_1	−0.018	−0.097	−0.049	−0.047	0.306***	−0.077	−0.025
		0	178.2		174.5	180.5	0.03	0.81	0.20	0.19	8.07	0.51	0.05
*qMulti6.06*	6.06	130.	PHM16607_11	6.88	PHM597_18	PZB01569_7	−0.027	0.108	0.010	0.178*	−0.064	0.111	0.045
		5	160.2		157.9	160.7	0.07	1.11	0.01	3.01	0.39	1.17	0.02
*qMulti8.02*	8.02	48.0	*ZmPSTOL8.02*	15.76	*ZmPSTOL8.02*	PHM1978_111	−0.252**	0.164	−0.243**	−0.184*	−0.177*	−0.095	−0.239**
			13.3		13.3	21.8	4.14	1.76	3.83	2.20	2.04	0.59	3.71
*qMulti9.04*	9.04	27.5	PHM13183_12	20.30	PZA0225_8	PZB01358_1	−0.022	−0.135^+^	−0.079	−0.286***	0.083	−0.164^+^	−0.148^+^
			104.7		104.5	106.8	0.04	1.71	0.58	7.67	0.65	2.53	2.05
*qMulti10.03*	10.03	38.0	PHM1155_14	19.96	PHM1812_32	PZA01877_2	−0.180*	−0.106	−0.240**	−0.403*** 15.17	−0.047	−0.171*	−0.013
			62.1		47.7	77.5	3.03	1.06			0.21	2.74	0.02
					^f^R^2^_T_		26.17	35.54	24.51	33.53	34.04	23.41	27.28

^a^QTLs are named using the “multi”, indicating that were detected using MT-MIM, followed by their genomic position in bin

^b^cM and Mbp indicate the marker position in centiMorgans and in mega base pairs at maximum LOD value

^c^Flanking markers are based on −1.5 LOD support interval

^d^Effect measured as standard deviation from the progeny mean: Positive values indicate that L3 carries the allele for an increase in the trait, and negative values indicate that L22 contributes the allele for an increase in the trait. Effect significance based on *p-values* estimated via score statistics resampling (^+^ *p* < 0.1, *p < 0.05, **p < 0.01, ***p < 0.001)

^e^R^2^: Ratio of the genotypic variance of the QTL effect to the phenotypic variance, times 100

^f^R^2^_T_: genotypic variance of the full model (including epistasis shown in Table 4)

MIM models for individual traits detected seven QTLs controlling root length, root diameter, surface area of fine roots, and root:shoot ratio. The proportion of the phenotypic variance explained by each QTL (R^2^) ranged from 6.84 % (*qRD4.05*) to 15.12 % (*qSA2_10.03*). The magnitude of QTL effects ranged from 0.270 standard deviations from the progeny mean (sd) to −0.393 sd (Table 3).

The MT-MIM analysis revealed the presence of ten QTLs with R^2^ ranging from 2.04 % (*qMulti8.02* for R:S) up to 15.17 % (*qMulti10.03* for SA2). The highest additive effect was also observed for *qMulti10.03* for SA2 (−0.403 sd) (Table 4).

LOD estimates for the MT-MIM model were higher and the confidence intervals were narrower than those for the individual MIM models, suggesting superior statistical power of MT-MIM compared with the MIM models applied to each trait individually (Fig. 2). Despite these differences, the MT-MIM and MIM models were coincident in revealing QTLs at bins 1.03, 1.07, 3.06, and 10.03, whereas QTLs at bins 1.06, 2.08, 3.04, 6.06, 8.02, and 9.04 were only revealed using the MT-MIM model. Conversely, QTLs at regions 4.05, 7.02, and 8.05 were only detected by MIM models. Therefore, combining the results of MIM and MT-MIM analyses conveyed the most accurate information regarding the genetic architecture of the traits under investigation in this particular study.

Using a simulation, Silva *et al*. [35] showed that when a QTL affects only a small subset of the traits, the MT-MIM model might have a lower power than MIM models to identify this QTL due to a greater genome-wide threshold for the MT-MIM model. This may be the reason why MT-MIM failed to identify QTLs at regions 4.05, 7.02, and 8.05. Although the MT-MIM LOD profile revealed peaks at these regions, the values were not statistically significant according to the score threshold employed.

The additive main effect of QTLs detected by MIM and MT-MIM had both positive and negative signs, confirming the contribution of favorable alleles coming from both parental lines for most of the traits analyzed (Tables 3 and 4). Additionally, five epistatic interactions were identified using the MT-MIM model, including some with magnitudes comparable to the main additive effects (Table 5). No epistatic effect was detected based on single trait analysis. Taken together, the additive and epistatic effects on the MT-MIM model explained between 23.41 % and 35.54 % of the phenotypic variance for each trait (Tables 4 and 5).

**Table 5:** Epistatic interactions for root morphology traits, total seedling dry weight and P content evaluated in low-P conditions

Interactions	Interaction effect^a^ / R^2^ (%)^b^						
	RL	RD	SA	SA2	R:S	TDW	Pcont
*qMulti1.03* X *qMulti1.07*	−0.060	0.067	−0.078	−0.183*	0.054	−0.053	−0.005
	0.33	0.41	0.56	3.07	0.27	0.25	0.03
*qMulti1.03* X *qMulti10.03*	−0.009	−0.029	−0.029	−0.141^+^	0.148*	−0.123	−0.235**
	0.01	0.08	0.08	1.85	2.04	1.41	5.12
*qMulti1.07* X *qMulti9.04*	0.324***	−0.358***	0.280**	0.127	−0.108	0.196*	0.067
	9.02	11.00	6.76	1.38	1.00	3.31	0.39
*qMulti2.08* X *qMulti10.03*	−0.071	0.179**	−0.013	0.115	0.166*	0.028	0.077
	0.45	2.86	0.02	1.18	2.47	0.07	0.53
*qMulti8.02* X *qMulti9.04*	0.231**	−0.150	0.254*	0.188^+^	−0.221*	0.307**	0.265**
	3.24	1.37	3.93	2.16	2.99	5.74	4.29

^a^Effects measured as standard deviation from the progeny mean; Positive values indicate that L3 carries the allele for an increase in the trait, and negative values indicate that L22 contributes the allele for an increase in the trait. Interaction effect significances based on *p*-values were estimated via score statistics resampling (^+^ *p* < 0.1, *p < 0.05, ** *p* < 0.01, ***p < 0.001)

^b^R^2^: Ratio of the genotypic variance of the QTL effect to the phenotypic variance, times 100

Traits: root length (RL), root average diameter (RD), root surface area (SA), surface area of fine roots (SA2), total seedling dry weight (TDW), root:shoot ratio (R:S) and total P content (Pcont)

### ZmPSTOL predicted proteins share a conserved serine/threonine kinase domain with OsPSTOL1

Using OsPSTOL1 [GenBank: BAK26566] as a query, six predicted proteins were selected on the maize genome, sharing more than 55 % amino acid sequence identity. The genes encoding these proteins were predicted to be located on chromosomes 3, 4 and 8, and named according to their genetic position in bin (Table 6).

**Table 6:** Maize candidate genes sharing more than 55 % amino acid sequence identity to OsPSTOL1

Predicted gene	Gene ID	Physical position (bp)	Identity (%)	Coverage (%)	E-value
GRMZM2G412760	*ZmPSTOL3.04*	Chr3: 20,172,140	55	99	5.1e-104
GRMZM2G448672	*ZmPSTOL3.06*	Chr3: 206,918,421	66	97	4.7e-186
AC193632.2_FG002	*ZmPSTOL4.05*	Chr4: 39,792,602	69	95	2.0e-105
GRMZM2G172396	*ZmPSTOL8.02*	Chr8: 13,267,001	55	99	9.6e-123
GRMZM2G451147	*ZmPSTOL8.05_1*	Chr8: 152,043,859	70	97	3.4e-131
GRMZM2G164612	*ZmPSTOL8.05_2*	Chr8: 152,100,275	70	97	2.3e-127

**Fig. 3:**
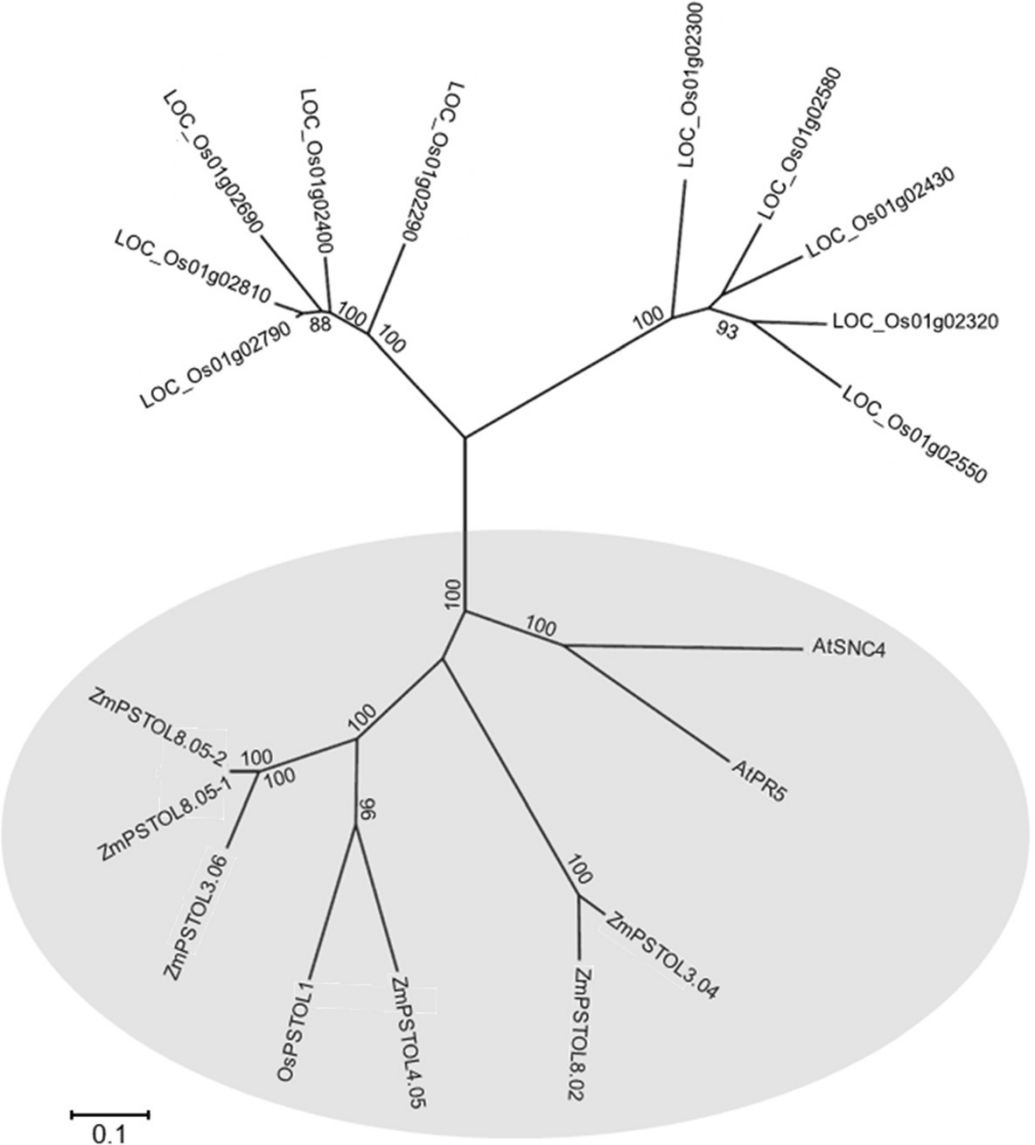
Phylogenetic tree of predicted serine/theronine receptor-like kinases from maize, rice and *Arabidopsis thaliana*. The rice PSTOL1, the six maize proteins sharing more than 55 % sequence identity to OsPSTOL1, PR5K and SNC4 from *Arabidopsis thaliana* were grouped separately from other rice kinases, and are highlighted. Numbers on branches are bootstrap values for the percentage of coincidence (%) inferred from 1,000 replicates. Only percentage values higher than 50 % are shown

A phylogenetic analysis revealed that the six predicted ZmPSTOL proteins clustered together with PSTOL1 from rice, SNC4 and PR5 from *Arabidopsis* (circled in Fig. 3), which were classified as LRK10L-2 subfamily of serine/threonine receptor-like kinases by Gamuyao *et al*. [40]. In a detailed alignment of structural predictions, all maize PSTOL-like proteins shared conserved ATP-binding and serine/threonine protein kinase domains with OsPSTOL1 (Additional file 3: Figure S2). A distinct glycosyl hydrolase domain was predicted for ZmPSTOL4.05. The maize proteins ZmPSTOL4.05, ZmPSTOL8.02, ZmPSTOL8.05_1 and ZmPSTOL8.05_2 were classified as receptor-like kinases (RLKs), which are characterized by the presence of a transmembrane domain for signal perception and an intracellular kinase domain [48, 49]. In contrast, the proteins ZmPSTOL3.04 and ZmPSTOL3.06 contained the intracellular kinase domain but lacked the transmembrane domain similarly to OsPSTOL1 [40], and thus were classified as receptor-like cytoplasmic kinases (RLCKs) [49].

### *ZmPSTOL* candidate genes have distinct expression patterns

**Fig. 4:**
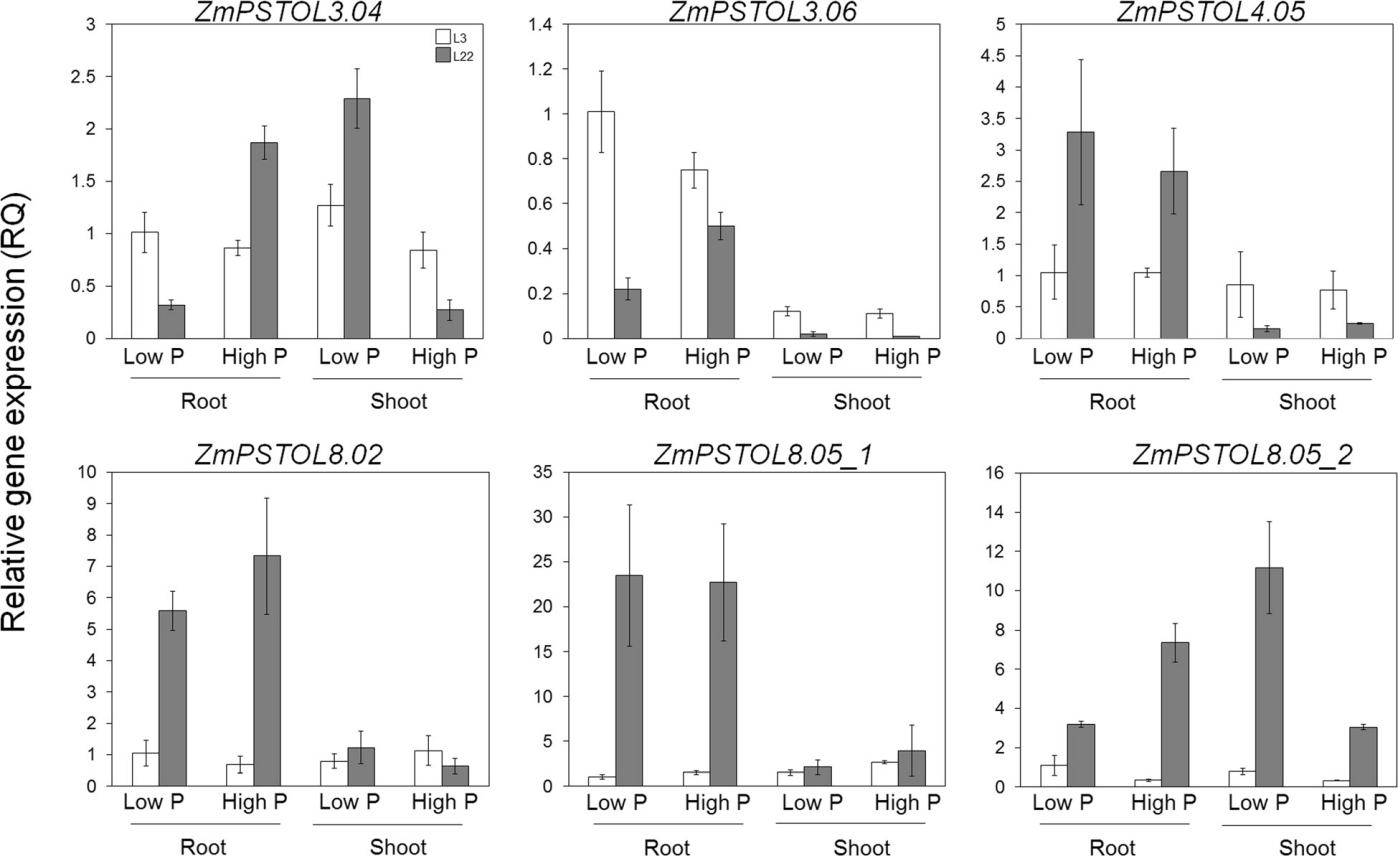
Expression profiles of the *ZmPSTOL* genes. The expression of the maize candidate genes are presented as relative gene expression (RQ) evaluated in roots and shoots of maize seedlings of the two parental lines L3 (P-efficient) and L22 (P-inefficient) grown under two levels of P (2.5 and 250 μM) after 13 days of treatment. Error bars indicate the standard errors of three technical replicates composed of three seedlings each

The expression analyses revealed that *ZmPSTOL4.05*, *ZmPSTOL8.02* and *ZmPSTOL8.05_1* were highly and consistently expressed in the roots of the P-inefficient genotype (L22) under low (2.5 μM) and high (250 μM) P conditions, but were not responsive to P in either L22 or L3 (Fig. 4). *ZmPSTOL3.06* was preferentially expressed in the roots of the P-efficient line (L3) with lower expression under high-P compared to the low-P concentration, and induced by high-P in roots of L22 (Fig. 4). The expression of *ZmPSTOL3.04* and *ZmPSTOL8.05_2* was induced in the root and repressed in the shoot of L22 under high-P concentration, but were not differentially expressed in L3 (Fig. 4). Additionally, the expression pattern of *ZmPSTOL3.04* and *ZmPSTOL8.05_2* in roots may reflect the total P content in the seedling, whereas the expression in shoots could be negatively associated with the total P content in L22 (Additional file 4: Table S2).

## Discussion

### Complex inheritance of root traits, seedling dry weight and P content in maize

Two distinct and powerful statistical models for QTL mapping (MIM and MT-MIM) were applied to dissect root morphology traits, total seedling dry weight, root:shoot ratio and P content in tropical maize RILs. The QTLs identified using the MIM model explained from 6 to 15 % of the total phenotypic variance for each trait, which was similar to the QTLs previously mapped for root traits and P efficiency indices in maize [25–27, 29, 31]. However, the proportion of phenotypic variance explained by all QTLs and their epistatic interactions using MT-MIM ranged from 23.4 to 35.5 %, which was higher than in previous studies. To the best of our knowledge, the present study demonstrates the first QTL mapping of root morphology traits, seedling biomass and P content in maize using the MT-MIM model, confirming that complementary information can be generated when this strategy is combined with single trait MIM analyses, as previously suggested by Silva *et al*. [35].

The genetic complexity of these traits was highlighted by the epistatic interactions among QTLs that showed effects of magnitudes comparable to those of main effects. The significant contribution of epistatic interactions was also detected by other authors for root traits in nutrient solution [25–27, 50] and for P-use efficiency indices under field conditions [30, 51].

QTL mapping revealed that both parents contributed favorable alleles for most of the traits evaluated, which possibly leads to transgressive segregation. The parents L3 and L22 were also shown to donate favorable alleles for P acquisition efficiency and P-use efficiency based on grain yield, when these RILs were backcrossed with both parental lines and evaluated in low-P soil [51]. The occurrence of segregating progenies with extreme phenotypes, out of the parental range, has been detected in plants subjected to different abiotic stresses under field or nutrient solution [50, 52, 53]. Transgressive segregation was also observed in maize RILs derived from a cross between Mo17 and B73 for the length and number of lateral and seminal roots [25, 27] and for root hair length [26].

The QTL mapping results strongly reflected the phenotypic correlations among the target traits. The high correlation between root length and root surface area (*r* = 0.98) reflected the coincidence in position and sign of the QTLs detected based on MT-MIM (*qMulti1.07*, *3.04*, *8.02* and *10.03*; Table 4). Additionally, two significant QTLs for root diameter were either mapped to unique regions or with opposite signs compared with other root traits, corroborating the negative correlations of these traits. Moreover, the importance of the root surface area to seedling dry weight and P content was supported by the presence of multi-trait QTLs that significantly affected these traits (*qMulti1.07*, *8.02* and *10.03*; Table 4), which could be a result of determinant genes with pleiotropic effects or the presence of linked genes.

### Coincidence of QTLs for root morphology in the seedling stage with QTLs for grain yield

Three genes previously associated with root morphology in maize (*Rth1*, *Bk2l3* and *Rtcs*) were mapped to chromosome 1, but did not overlap with any mapped QTLs (Fig. 2). Even though early root growth enhancement has not always led to superior yield performance in the field [54], a large number of QTL studies have indicated that some genomic regions consistently affect root morphology traits during the seedling stage and agronomic performance under different environments. A similar situation was also found for three QTL regions on chromosome 1 in our current study, which were coincident with QTLs previously reported for early root traits and for yield components in the field.

*qMulti1.03* associated with root diameter, total seedling dry weight and root:shoot ratio overlapped with the QTL influencing seminal root length and weight [50], primary axile root diameter [55], daily elongation rate of axile roots [56] and total length of second-order lateral roots [57] in nutrient solution. This region was coincident with QTLs for drought tolerance index [50], grain yield, kernel number and weight in low-P soil [58]. This genomic region was also detected in a meta-analysis for low-P tolerance in maize as the consensus cQTL1-2 [59].

*qMulti1.06* was detected based on the multi-trait MIM model and was significantly associated only with the total P content in the seedling (Table 4 and Fig. 2). A major QTL at bin 1.06 was associated with root traits in nutrient solution, grain yield under well-watered and water-stressed conditions [50], and root-pulling resistance in adult plants [60]. Due to the consistency in the effects of this genomic region on root traits and grain yields in different studies, this QTL was named *root-yield-1.06*, and validated as constitutively affecting roots, agronomic features and grain yield under different water regimens [61]. A QTL for P utilization efficiency (*qPUTIL1*) based on grain yield under low-P soil also overlapped in this region [51].

The third QTL region on chromosome 1 was mapped at bin 1.07 spanning from 214 to 223 Mbp (*qMulti1.07*), which was associated with root length, root surface area, surface area of fine roots, total seedling dry weight and root:shoot ratio. This region was coincident with a cluster of QTLs named Ax-2 that controlled the root numbers and lengths identified in a meta-analysis combining 15 QTL studies [54]. QTLs for grain yield and drought tolerance index were also mapped to this genomic region [50].

The association of root morphology QTLs in early stages of plant development with yield performance, including the validation of the *root-yield-1.06*, suggested that at least some of these genomic regions can be further used in marker-assisted selection to improve yield stability under drought and other mineral stresses in maize.

### *ZmPSTOL* genes co-localized with QTLs for root morphology, biomass accumulation and P content

The QTL mapped using MT-MIM (*qMulti8.02*) that was associated with root length, root surface area, root:shoot ratio and P content co-localized with *ZmPSTOL8.02* (Fig. 2). This *ZmPSTOL* candidate gene was highly expressed in the roots of L22 (Fig. 4), the donor line of the favorable QTL alleles for all traits mentioned above. In rice, the overexpression of *OsPSTOL1* enhanced total root length and root surface area in transgenic seedlings in nutrient solution as well as grain yield of transgenic varieties cultivated in P-deficient soils [40]. According to these authors, the larger root system contributed to a significant increase in the uptake of nutrients such as phosphorus, nitrogen and potassium in transgenic rice lines overexpressing *OsPSTOL1*. The sequence similarity and conserved domains of these protein kinases from rice and maize combined with the evidences shown here suggest that *ZmPSTOL8.02* could be one of the genes underlying *qMulti8.02*, sharing similar functions in root development and P acquisition efficiency in maize to *OsPSTOL1* in rice. Additionally, QTLs associated with seminal root number in high-P levels [27], shoot dry weight [32] and primary root length [56] overlapped with *qMulti8.02*, confirming that genes controlling root and shoot development in this genomic region are also expressed in other genetic backgrounds.

The other three *ZmPSTOL* genes co-localized with single trait QTLs for root length (*ZmPSTOL8.05_1*), root diameter (*ZmPSTOL4.05*), and root:shoot ratio (*ZmPSTOL3.06*). These genes were preferentially expressed in the roots of the donor line that contributes positive alleles for the respective QTLs under low- and high-P conditions.

*ZmPSTOL8.05_1* was mapped to 117 cM on chromosome 8, flanking *qRL8.05*. Additionally, a weak LOD peak for total P content was coincident with this candidate gene (Fig. 2). The MIM model based on score as a significance threshold was not able to detect this QTL, but a minor effect QTL was detected using the Bayesian Information Content threshold with LOD 1.8 and explaining 6.5 % of the total variance for the total P content in the seedling (data not shown). This genomic region also harbored QTLs explaining 5 to 6 % of the phenotypic variance for P acquisition efficiency based on grain yield [51], indicating that this genomic region consistently contributed to P acquisition during the seedling and adult plant stages. Additionally, QTLs in this genomic region were mapped for root length and grain yield under field conditions [32], root length and root dry weight in nutrient solution [26, 50, 62]. The coincidence of QTLs for root traits and for grain yield from different studies associated with the superior expression of *ZmPSTOL8.05_1* in roots under low-P are highly compatible with the role of its putative homolog (*OsPSTOL1*) in rice.

*ZmPSTOL3.06* was mapped to 169.6 cM on chromosome 3 within the confidence interval of *qRS3.06* for root:shoot ratio (Fig. 2). This genomic region spans bin 3.06 that harbored QTLs for root traits in a meta-analysis using 15 QTL studies in nine maize mapping populations [54]. This candidate gene was highly expressed in roots cultivated with both P levels of the P-efficient line L3 (Fig. 4), which contributed with positive alleles for the root:shoot ratio QTL. *ZmPSTOL3.06* had the lowest e-value with *OsPSTOL1* (Table 6) and its predicted protein lacks the transmembrane domain, similarly to OsPSTOL1 (Additional file 3: Figure S2). This combined information makes this predicted gene also a candidate to *OsPSTOL1* homolog in maize.

*ZmPSTOL4*.05 was coincident with the *qRD4*.05, with L22 donating the allele that reduced the root diameter. *ZmPSTOL4*.05 was highly expressed only in roots of the donor line, L22, cultivated in both P availability. Under P starvation conditions the root diameter decreases, while the root surface area increases, enabling the root system to explore a larger volume of soil [11]. Thus, as observed in the path analysis, fine roots are an important component to improve the root surface area, which played a strong contribution to total seedling dry weight and to P content in maize seedlings cultivated in low-P conditions. A QTL controlling the plasticity of lateral root number (i.e., change in the lateral root number in response to P availability) in hydroponics [26] was also mapped at this same region, suggesting that genes harboring in this genomic region may control root traits across different populations.

The finding that *ZmPSTOL* genes were preferentially expressed in the roots of the lines that contributed the allelic enhancing root traits, seedling dry weight and P content indicate that at least *ZmPSTOL3.06*, *ZmPSTOL4.05*, *ZmPSTOL8.02* and *ZmPSTOL8.05_1* may have a functional relationship with root morphology and/or with P acquisition in maize. Considering the role of *PSTOL1* genes in rice and sorghum, it could be expected that genes encoding important adaptive traits would be shared among rice, sorghum and maize, such as the case for the major Al tolerance gene in sorghum (*SbMATE*) [63] that was found to be functionally conserved in maize (*ZmMATE1*) [64] and rice (*OsFRDL4*) [65].

## Conclusions

Comprehensive QTL analyses revealed important regions associated with root traits, seedling dry weight and P content in maize under low-P concentration. Using the multiple trait-multiple interval mapping model, these QTLs explained a larger extent of the phenotypic variance for the target traits compared with previous studies. The complementary genomic regions identified using both models jointly offered putative targets for molecular breeding aiming to improve P acquisition efficiency in maize. Additionally, this study identified new maize candidate genes sharing high identity with *OsPSTOL1* that were preferentially expressed in the roots and co-localized with QTLs for root morphology and P acquisition related traits.

## Material and methods

### Mapping population

The segregating population was composed by 145 maize recombinant inbred lines (RILs) derived from a bi-parental cross of lines L3 (P-efficient) and L22 (P-inefficient). F_1_ plants were self-pollinated and individual F_2_ plants were advanced for seven cycles of selfing by single seed descent, after which seeds were bulked for multiplication. The parental lines and the population were developed at Embrapa Maize and Sorghum (Brazil, latitude 19_270S and 716 m above sea level). The parental lines were previously characterized as contrasting for P-use efficiency under low and high P conditions in the field [45] and for root morphology traits [46].

### Quantitative analysis of root traits, seedling biomass accumulation and P content using a paper pouch system

The mapping population and parents were evaluated in randomized complete block design with four biological replicates, each composed by three plants per pouch. Each biological replicate was evaluated in an identical but independent experiment performed on a seven-day interval. Maize seeds were surface sterilized with 0.5 % (v/v) sodium hypochlorite for 5 min and germinated in moistened germination paper rolls. After four days, uniform seedlings were transferred to moist blots in paper pouches after removing the endosperm to eliminate seed reserves [46]. A modified Magnavaca nutrient solution [66] containing 2.5 μM P was replaced every three days and the pH was maintained at 5.65. Each container was filled with 5 l of nutrient solution with the bottom 3 cm of the pouches immersed in the solution. The containers were maintained in a growth chamber with a 12 h photoperiod at 27/20 °C day/night temperatures and 330 μmol m^−2^ s^−1^ of light intensity. After 13 days, root images were captured using a digital photography setup and analyzed using RootReader2D (http://www.plantmineralnutrition.net/rr2d.php) and WinRHIZO (http://www.regent.qc.ca/assets/winrhizo_about.html) software according to de Sousa *et al*. [46]. The total root system, which includes all together primary, seminal and initial adventitious roots, was evaluated for total root length (RL) (cm), average root diameter (RD) (cm^3^), total root surface area (SA) (cm^2^) and surface area of fine roots (SA2) (1.0 < d ≤ 2.0 mm) (cm^2^).

Root and shoot tissues were dried separately at 65 °C in a forced-air oven until a constant weight was obtained to determine the root:shoot dry weight ratio (R:S) and total seedling dry weight (TDW). For P analysis, root and shoot tissues were subjected to nitric perchloric acid digestion [67]. The total P content in the seedling (Pcont) was calculated as the sum of the P content in each seedling component, which was the product of the dry weight and the P concentration in the root and shoot. As maize absorbs phosphate in its orthophosphate form (Pi), the P in the nutrient solution refers to phosphate, whereas the total P content in the seedling comprises both organic and inorganic P forms.

Analysis of variance (ANOVA), correlations between pairs of traits and path analysis were performed using the GENES software [68]. The phenotypic correlations were calculated based on the mean values. Broad sense heritability was estimated as ${h}^2={\widehat{\sigma}}_G^2/\left({\widehat{\sigma}}_G^2+{\widehat{\sigma}}_E^2\right)$ with ${\widehat{\sigma}}_G^2 = \left(M{S}_G\mathit{\hbox{-}}\ M{S}_E\right)/r$ and ${\widehat{\sigma}}_E^2=M{S}_E$, where ${\widehat{\sigma}}_G^2$ and ${\widehat{\sigma}}_E^2$ are the estimates of genetic and error variance, respectively; *MS*_*G*_ and *MS*_*E*_ are the genetic and error mean squares, respectively, and *r* is the number of replications.

For the path analysis [69], the five root traits (total root length, root average diameter, surface area and surface area of fine roots) were considered as independent variables *x*_*i*_ (*i* = 1, 2, …, 5). The total seedling dry weight and phosphorus content were considered as dependent variables *y*_*j*_ (*j* = 1, 2) in two distinct path analyses. The estimated path coefficient (*P*_*ij*_) was considered as the direct effect of variable *x*_*i*_ on *y*_*j*_. Indirect effects of *x*_*i*_ on *y*_*j*_ mediated by variable *x*_*i′*_ were calculated by multiplying the correlation between *x*_*i*_ and *x*_*i′*_ (*r*_*ii′*_) by *P*_*i′j*_. Root:shoot ratio was excluded from the analysis due to its contribution to both dependent variables.

### Linkage map

DNA was isolated from young leaves using the CTAB method [70]. Initially, 60 polymorphic SSR markers were genotyped in the RIL population according to [71]. A total of 332 SNPs (Single Nucleotide Polymorphisms) were genotyped in the population using Kompetitive Allele-Specific PCR or the KASP^TM^ assay (LGC Genomics, Teddington, UK). The sequences, genetic and genomic locations of SSR and SNP markers are available at the Maize Genetics and Genomics Database (http://www.maizegdb.org/data_center/locus).

The markers were tested for the expected segregation ratio of 1:1 using chi-square statistics (*p* < 0.05) corrected for multiple tests based on Bonferroni’s method. The linkage map was constructed using MapMaker/EXP 3.0 [72] considering a minimum LOD of 3.0 and a maximum frequency of recombination (*r*) of 0.4. The mapping function Kosambi [73] was used to convert recombination fractions into map distances. The final linkage map was drawn using Windows QTL Cartographer v 2.5 (http://statgen.ncsu.edu/qtlcart/WQTLCart.htm).

### QTL mapping

Phenotypic fitted values were obtained from the following statistical model adjusted for each single trait:

$$ {y}_{ij}=\mu +{B}_i+{G}_j+{\varepsilon}_{ij} $$

where *y*_*i*j_ is the phenotypic observation from the *i*^*th*^ (*i* = 1, …, 4) replication on the *j*^*th*^ genotype (*j* = 1, …, 145); *μ* is the phenotypic average; *B*_*i*_ is the effect of the *i*^*th*^ block; *G*_*j*_ is the *j*^*th*^ genotype; and *ε*_*ij*_ is the residual associated with the *y*_*ij*_ observation. We tested the fit of two models by first assuming that the residuals were normally distributed with constant variance, *ε*_*ij*_ *~ N(0, σ*^*2*^*)* and by second considering the heteroscedasticity, *ε*_*ij*_ *~ N(0, σ*_*j*_^*2*^*)*. Both models were fitted using the *gls* function from the *nlme* R package [74] and compared using the *ANOVA* function from the same package. For each trait, the fitted phenotypic values were extracted from the model with the best fit. Each replicate was composed by three plants that were bulked for all laboratorial analyses.

Due to the large variability in the absolute values, all phenotypic fitted values were standardized to achieve unity as standard deviations and zero means as follows:

$$ {z}_{it}=\frac{y_{it} - {\mu}_t}{\sigma_t}, $$

where *z*_*it*_ is the standardized observation of trait *t* (*t* = 1, 2, …, 7) on subject *i*; *y*_*it*_ is the observation of trait *t* on subject *i*; *μ*_*t*_ is the average of trait *t*; and *σ*_*t*_ is the standard deviation of trait *t*.

For the joint QTL analysis, a multiple trait-multiple interval mapping (MT-MIM) model was evaluated as previously described [35]. The complete model was fitted using the following equation:

$$ {z}_{ti}={\mu}_t+{\displaystyle \sum_{r= 1}^m{\beta}_{tr}}{x}_{ir}+{\displaystyle \sum_{r<l}^p{W}_{trl}}{x}_{ir}{x}_{il}+{\varepsilon}_{ti}, $$

where *z*_*ti*_ is the standardized observation of trait *t* on subject *i*; *μ*_*t*_ is the intercept for trait *t*; the parameter *β*_*tr*_ has the genetic interpretation of the additive effect of QTL *r* on trait *t* (*r* = 1, 2, …, *m* QTLs included in the model); and the regressive variables *x*_*ir*_ represent the contrast coefficients codified according to the Cockerham model [33, 75] (i.e., *x*_*ir*_ is 1 for the dominant and −1 for the recessive homozygous). The third component on the right side of the model refers to a subset of the *p* pairwise interactions among QTLs previously included in the model, where *w*_*trl*_ is the epistatic effect between QTL *r* and QTL *l* on trait *t* and the random error *ε*_*ti*_ was assumed to be independent and identically distributed according to a multivariate normal distribution, with a mean vector of zero and a positive definite symmetric variance-covariance matrix ∑_*ε*_, i.e., *ε*_*ti*_ = *MVN*(*0*, ∑_*ε*_).

Multi-trait QTL mapping was initiated with a forward search for the main effect QTL using a grid of 1 cM in the genome and a 15 % genome-wide significance level. After three rounds of QTL search, the positions of all QTLs in the model were re-estimated as along with all other parameters in the model. After the inclusion of the main effects in the model, the forward search for epistasis was performed by testing all pairwise interactions among QTLs already included in the model, employing a 5 % genome-wide significance level. Only the epistatic effects that displayed at least one significant marginal effect were kept in the final model. The Haley-Knott regression [76] was used to estimate the model parameters, and the resampled score statistics [35, 77] were employed to obtain the empirical genome-wide threshold for the QTL mapping analysis. Using this approach, a final model was selected to calculate the proportion of phenotypic variance explained by all QTLs as the ratio between the genotypic variance of the QTL effect to the phenotypic variance times 100 (coefficient of determination R^2^), and the LOD profile along the chromosomes. The R^2^ values were estimated using the fitted full model, including non-significant QTL effects. The QTL confidence intervals were obtained using the drop 1.5-LOD support interval method with approximately 0.95 confidence levels [78].

Multiple interval mapping (MIM) analysis was performed for each single trait [33, 79] in a similar procedure to that performed for the joint analysis, considering *t* = 1. All QTL analyses were performed using R software (version 2.15.2) and a QTL mapping package named OneQTL that is under development by L.C. Silva.

### Identification of maize *PSTOL1* homologs

Using the OsPSTOL1 amino acid sequence [GenBank: BAK26566] we performed searches against the maize genome database (http://ensembl.gramene.org/Zea_mays/Info/Index) using BLASTp. Six predicted maize proteins with more than 55 % sequence identity to rice PSTOL1 were selected and aligned using ClustalX software version 1.83 [80]; the alignment included OsPSTOL1 and the Arabidopsis protein kinases SNC4 [81] and PRK5 [82]. The phylogenetic tree was constructed based on the maximum likelihood method with 1000 bootstraps [83] using MEGA software [84]. The protein domains were identified using the CDART (Conservative Domain Architecture Retrieval Tool (http://www.ncbi.nlm.nih.gov/Structure/lexington/lexington.cgi).

### Mapping candidate genes

Specific primers for the maize candidate genes were designed using Primer Blast (ncbi.nlm.nih.gov/tools/primer-blast/index.cgi). PCR reactions were performed using 30 ng of DNA, 0.2 mM of each dNTP, 2 mM of MgCl_2_, 10 ρmols of each primer, 5 % (v/v) dimethyl sulfoxide (DMSO) and 1 U of Taq DNA polymerase (Invitrogen, Carlsbad, CA). The amplification profile included an initial step of 95 °C for 1 min, followed by 35 cycles of denaturing at 94 °C for 1 min, annealing at 58 to 60 °C for 30 s, depending on the primers, and extension at 72 °C for 1 min. The amplification products were treated with ExoSAP-IT reagent (USB Corporation, Cleveland, OH) and sequenced using the BigDye Terminator v3.1 cycle sequencing kit on an ABI PRISM 3100 genetic analyser (Applied Biosystems, Foster City, CA) to identify polymorphisms between the parental lines.

Sequence-tagged site (STS) markers were developed to map genes previously associated with root morphology in maize. For the *roothairless* gene (*Rth1*) [22], a 22-bp indel was amplified using the primers 5′-TTGCCCACGGCTGGCAAGAG-3′ and 5′- GGCTCTGTAGCACGCCCCTC - 3′ and resolved on a silver-stained polyacrylamide gel [85]. The same strategy was used for the *brittle stalk-2-like protein 3* gene (*Bk2l3*) [23], which was revealed after the amplification of a 15-bp indel using the primer pair: 5′-GCTGGTTAGATCCCCCGCCCA-3′ and 5′-GCACTGGAGCCACCGACACTG-3′. The *rootless concerning crown and seminal roots* gene (*Rtcs*) [24] was genotyped as a CAPS marker obtained after digestion of the amplification product of genomic DNA with the primers 5′-CGCGCCATAGCCCGCAGTAA-3′ and 5′-GATTGGCACGGGCCGGTCAG-3′ with the restriction enzyme *Aci*I and was visualized on a silver-stained polyacrylamide gel [85].

Cleaved amplified polymorphic sequence (CAPS) markers were developed for the other candidate genes. For *ZmPSTOL3.04* the PCR product amplified using the primers 5′-ACGGGGCTTGGAGGCACATG-3′ and 5′-TGAGACCGCGTGGGGAAGGG-3′ was digested with the restriction enzyme *Stu*I. The polymorphism for *ZmPSTOL8.02* was obtained after digestion with *Rsa*I of the genomic fragment amplified with the primers 5′-TGACTGGTGCCAGAGGTACGC-3′ and 5′-TGCATACAAGGGACTGCTTCGGA-3′. CAPS markers were resolved on silver stained polyacrylamide gels [85]. The images were digitally captured using a Nikon digital camera.

*ZmPSTOL3.06* was mapped based on the presence and absence of the amplification product using the primers 5′-AAGGGCGTCCAACCGCCTTG-3′ and 5′-TTGTTGGCCGGTCCGTTGGG-3′ on a 1 % (w/v) agarose gel stained with ethidium bromide.

A G/A SNP was revealed after sequencing the amplified product obtained using the primer pair 5′-CCGCTACGCCTTGGTTGCCA-3′ and 5′-CGCCGTAGTTAGCGGAGCCG-3′ to map *ZmPSTOL4.05,* the primer pair 5′-AGCCTCCACGATGGCCGACA-3′ and 5′-TGCATTTGTGTGACCTGGAA-3′ to map *ZmPSTOL8.05_1*, and the primers 5′- TCCACGGCCGACAGGTAGCA-3′ and 5′-GCTCAAGAGAACTCAGGGTGGC-3′ to map *ZmPSTOL8.05_2*.

### Gene expression analysis

The expression profiles of the candidate genes were assessed in the roots and shoots of the L3 and L22 genotypes harvested after 13 days in modified Magnavaca’s nutrient solution containing low (2.5 μM) and high (250 μM) P. Total RNA was extracted from a bulk of three plants using the RNeasy Plant Mini kit (Qiagen, Valencia, CA), and 1 μg of total RNA pretreated with DNase I was used for cDNA synthesis using the High Capacity cDNA Reverse Transcription kit (Applied Biosystems, Foster City, CA) according to the manufacturer’s instructions. Gene expression was determined by quantitative PCR (qPCR-RT) using SYBR Green I and TaqMan assays in the ABI Prism 7500 Fast System (Applied Biosystems, Foster City, CA). Primers were designed for all target genes using Primer Express Software (Applied Biosystems, Foster City, CA), and 18S rRNA was used as an endogenous constitutive control (Additional file 5: Table S3). With the exception of *ZmPSTOL4.05*, two primers pair were designed for each gene to confirm their expression pattern. However, only the expression profile obtained with the primer pairs highlighted in bold in the Additional file 5 are shown. The relative gene expression was calculated using the 2^-ΔΔCT^ method [86], with three technical replicates and L3 roots under low-P conditions as a calibrator. Variance analysis of the gene expression and total P content was performed using GENES software [68].

### Availability of supporting data

http://purl.org/phylo/treebase/phylows/study/TB2:S17800.

## Additional files

Additional file 1: Table S1. Trait means, coefficients of variation (CV), genetic variances (*σ*_*G*_^*2*^), environmental variances (*σ*_*E*_^*2*^) and heritability estimates (*h* ^*2*^) in 145 RILs derived from a cross between maize lines L3 and L22. Additional file 2: Figure S1. Genetic linkage map including 292 markers in a maize RIL population derived from a cross between L3 and L22. For each chromosome (chr), marker names are indicated on the right and the genetic distance in centimorgans (cM) is on the left. The colored bars indicate the position of QTLs for root traits, total plant dry weight, root:shoot ratio and P content (for details see Fig. 3, Tables 3 and 4). Additional file 3: Figure S2. Alignment of the OsPSTOL1 and six maize serine/theronine receptor-like kinases highlighting the predicted domains. Letters in green indicate the glycosil hydrolase domain and in red, the transmembrane domain. The kinase domain is represented by a gray background, whereas the ATP-binding site is highlighted with black background with white letters and the Serine/Threonine protein kinase active site with yellow background. Additional file 4: Table S2. Total P content in the seedling and relative expression of *ZmPSTOL* genes in shoots and roots of both parental lines in low- and high-P concentration. Additional file 5: Table S3. Primers used for gene expression analyses.

## Abbreviations

BIC: Bayesian information content
bp: Base pair
CAPS: Cleaved amplified polymorphic sequence
CDART: Conservative domain architecture retrieval tool
CI: Confidence interval
cM: centiMorgan
CTAB: Cetyl trimethyl ammonium bromide
ICP: Inductively-coupled argon
indel: Insertion/deletion
KASP: Kompetitive allele-specific PCR
LOD: Likelihood of odds
Mbp: Mega base pairs
MIM: Single trait-multiple interval mapping
MT-MIM: Multiple traits-multiple interval mapping
μM: micro Molar
mM: mili Molar
Pcont: Total phosphorus content in the seedling
PCR: Polymerase chain reaction
P: Phosphorus
PSTOL1: Phosphorus-starvation tolerance 1
qPCR-RT: Quantitative real-time PCR
QTL: Quantitative trait loci
RD: Average of root diameter
RIL: Recombinant inbred line
RL: Total root length
R:S: Root:shoot dry weight ratio
SA: Total root surface area
SA2: Surface area of fine roots
sd: Standard deviations from the progeny mean
SNP: Single nucleotide polymorphism
SSR: Simple sequence repeats
STS: Sequence-tagged site
TDW: Total seedling dry weight
v/v: Volume per volume
w/v: Weight per volume
