# Supplementary material for: Multiple interval QTL mapping and searching for PSTOL1 homologs associated with root morphology, biomass accumulation and phosphorus content in maize seedlings under low-P
Source: BMC Plant Biol. 2015 Jul 7;15:172. doi: 10.1186/s12870-015-0561-y (PMC4492167; doi:10.1186/s12870-015-0561-y)
Supplement: Additional file 1: Table S1. — Trait means, coefficients of variation (CV), genetic variances (σ G2), environmental variances (σ E2) and heritability estimates (h 2) in 145 RILs derived from a cross between maize lines L3 and L22. [file 12870_2015_561_MOESM1_ESM.docx]

**Additional File 1: Table S1** Trait means, coefficients of variation (CV), genetic variances ($\sigma_{G}^{2}$), environmental variances (**$\sigma_{E}^{2}$**) and heritability estimates (*h^2^*) in 145 RILs derived from a cross between maize lines L3 and L22

| **Trait** | **Mean** | **CV(%)** | **$\sigma_{G}^{2}$** | $\sigma_{E}^{2}$ | ***h^2^*** |
| --- | --- | --- | --- | --- | --- |
| Root length (cm) | 142.06 | 26.2 | 1683.2 | 353.4 | 0.82 |
| Root diameter (cm^3^) | 0.88 | 7.4 | 0.004 | 0.001 | 0.79 |
| Root surface area (cm^2^) | 38.48 | 23.4 | 91.6 | 20.6 | 0.81 |
| Surface area of fine roots (cm^2^) | 14.50 | 24.3 | 13.5 | 3.2 | 0.81 |
| Total seedling dry weight (g) | 0.09 | 20.2 | 0.004 | 0.0001 | 0.81 |
| Root:shoot ratio | 0.79 | 20.5 | 0.012 | 0.006 | 0.65 |
| Total P content (mg) | 0.65 | 17.4 | 0.012 | 0.003 | 0.75 |
