# Supplementary figures and images for: Multiple interval QTL mapping and searching for PSTOL1 homologs associated with root morphology, biomass accumulation and phosphorus content in maize seedlings under low-P
Source: BMC Plant Biol. 2015 Jul 7;15:172. doi: 10.1186/s12870-015-0561-y (PMC4492167; doi:10.1186/s12870-015-0561-y)

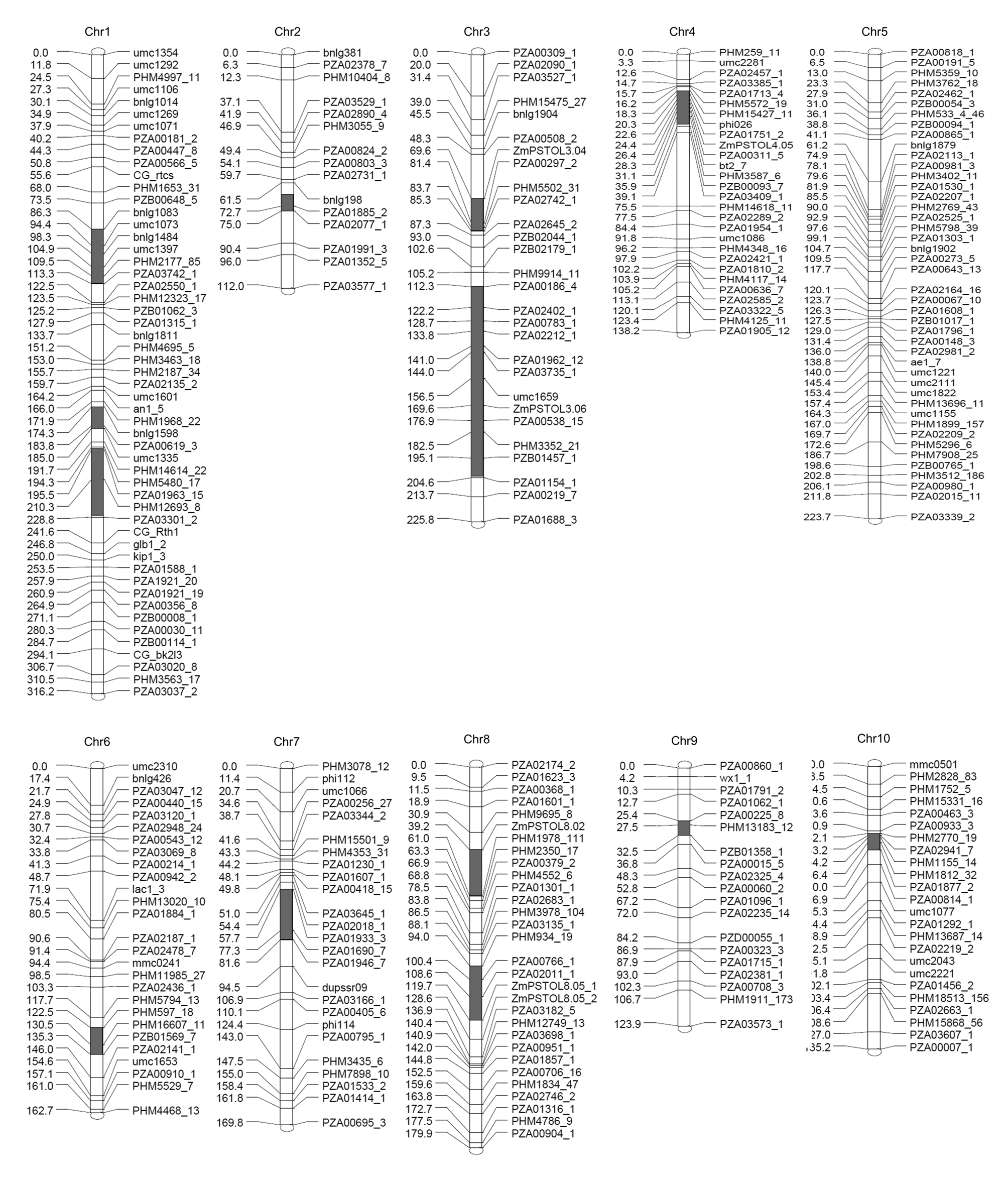

Supplement: Additional file 2: Figure S1. — Genetic linkage map including 292 markers in a maize RIL population derived from a cross between L3 and L22. For each chromosome (chr), marker names are indicated on the right and the genetic distance in centimorgans (cM) is on the left. The colored bars indicate the position of QTLs for root traits, total plant dry weight, root:shoot ratio and P content (for details see Fig. 3, Tables 3 and 4). [file 12870_2015_561_MOESM2_ESM.tif]
