# Supplementary material for: Multiple interval QTL mapping and searching for PSTOL1 homologs associated with root morphology, biomass accumulation and phosphorus content in maize seedlings under low-P
Source: BMC Plant Biol. 2015 Jul 7;15:172. doi: 10.1186/s12870-015-0561-y (PMC4492167; doi:10.1186/s12870-015-0561-y)
Supplement: Additional file 3: Figure S2. — Alignment of the OsPSTOL1 and six maize serine/theronine receptor-like kinases highlighting the predicted domains. Letters in green indicate the glycosil hydrolase domain and in red, the transmembrane domain. The kinase domain is represented by a gray background, whereas the ATP-binding site is highlighted with black background with white letters and the Serine/Threonine protein kinase active site with yellow background. [file 12870_2015_561_MOESM3_ESM.docx]

OsPSTOL1 M-----------------------------------------------------------

ZmPSTOL3.04 M---------------ARLEPPLTRVV---------------------------------

ZmPSTOL3.06 M-----------------------------------------------------------

ZmPSTOL4.05 MACNRRRKLLCACLLLACAAPAAAAAVNISVYWGQNSNEGSLGQTCSSGRYALVAMAFLS

ZmPSTOL8.02 M---------------DMIVQPLFCTVDCIVH--------------------------IV

ZmPSTOL8.05_1 MAMG--------------------------------------------------------

ZmPSTOL8.05_2 MYVK------------------LEEVVHTEV----------------------------E

*

OsPSTOL1 ------------------------------------------------------------

ZmPSTOL3.04 ------------------------------------------------------------

ZmPSTOL3.06 ------------------------------------------------------------

ZmPSTOL4.05 TFGSGQTPVLNLAGHCDPASGGCTALAADIAACQARGVRVLLSIGGGAGSYNLSSASDAE

ZmPSTOL8.02 NFEKGN------------------------------------------------------

ZmPSTOL8.05_1 ------------------------------------------------------------

ZmPSTOL8.05_2 HMQEGHTEVL--------------------------------------------------

OsPSTOL1 ------------------------------------------------------------

ZmPSTOL3.04 ------------------------------------------------------------

ZmPSTOL3.06 ------------------------------------------------------------

ZmPSTOL4.05 SCPYPDASLGAALATGLFDHVWVQFYNNPGCEYQQKDGDGVANLAASWKAWTQSLPSSAS

ZmPSTOL8.02 ------------------------------------------------------------

ZmPSTOL8.05_1 ----------------------LDF-----------------------------------

ZmPSTOL8.05_2 -------------------RKKFDFH----------------------------------

OsPSTOL1 ------------------------------------------------------------

ZmPSTOL3.04 ---------------------------------------------------TGLGG----

ZmPSTOL3.06 ------------------------------------------------------------

ZmPSTOL4.05 VFLGLPASPAAAGSGYVPPDDLVSRVLPAVSGSANYGGLMLWNRY--YDNSTGYSARILS

ZmPSTOL8.02 ----------------------------------------------EIGDMMGTKGNGRK

ZmPSTOL8.05_1 -----------------------------SHGNSSIKGWGMWKFLPQGKNSPPTGSKRKE

ZmPSTOL8.05_2 ------------------PQQV------EGHTDVPRKKLEFWLGKLEVDHMKAARSKRNE

OsPSTOL1 ---------------------------------------------LLCQRA-----SKNA

ZmPSTOL3.04 --------------------------TWCQRERQEDNTNRQKLRFFLCKKT----SSSIQ

ZmPSTOL3.06 ----------------------------------------------------LPFKSKDE

ZmPSTOL4.05 -SNIIAGTAGVSGL---CAIIALAALMW----------------WYKRRYGMVIPWRRGV

ZmPSTOL8.02 -IPLIVSISVAASLLLPCIYV----LVW----------HRQKLEFFLCKKT----SSAIE

ZmPSTOL8.05_1 AAPIVGA-VAVAFL---CLVI----LTS----------------FLACRYGLLPFKSKNK

ZmPSTOL8.05_2 -GPIVGAVVAVAFL---CLVI----LTC----------------FLACRHGSLPFKSKNK

OsPSTOL1 P--RIESFLQKQE-TSNPKRYTLSEVKRMTKSFAHKLGRGGFGTVYKGSLPDGREIAVKM

ZmPSTOL3.04 E--NIEALISSYG-SLAPKRYKYSEVTKITSCINNKLGEGGYGVVFKGMLDDSRLVAVKF

ZmPSTOL3.06 P--RIESFLQKNG-NLHPKRYTYADVKRMTRSFTEKLGQGGFGAVYRGSLHDGRQVAVKM

ZmPSTOL4.05 S--GVESFLQKQGALLHPKGYTYSEVKRMTRSFAHKLGQGGYGAVYRGSMPDGREVAVKM

ZmPSTOL8.02 E--NIEALILAHG-SLAPKRYRYSEVTKITSSLNIKLGEGGYGMVFKGRLDDGRLVAVKF

ZmPSTOL8.05_1 PGTRIESFLQKNE-SIHPKRYTYADVKRMTKSFAVKLGQGGFGAVYKGSLHDGRQVAVKM

ZmPSTOL8.05_2 PGTRIESFLQKNESSIHPKRYTYADVKRMTKSFAVKLGQGGFGAVYKGSLH-GRQVAVKM

:*::: ** * ::*.::* .: ***.**:* *::* : .* :***:

OsPSTOL1 LKDT-KGDGEEFINEVAGISKTSHINVVNLLGFSLQGSKRALIYEYMPNGSLDRYSFGDS

ZmPSTOL3.04 LHDS-KGDGEEFVNEVMSIGRTSHINIVSLFGFCLEGSKRALIYEYMPNGSLDKYIY---

ZmPSTOL3.06 LKDT-KGDGEEFMNEVASISRTCHVNIVTLTGFCLQGSKRALVYEYMPNGSLERYAF---

ZmPSTOL4.05 LTGMLEGDGEEFMNEVASISRTSHVNIVTLVGYCLQGPKRALLYEYMPNGSLERYTF--G

ZmPSTOL8.02 LHDS-KGDGEEFVNEVMSIGRTSHINIVSLFGFCLEGSKRALIYEYMSNGSLDKYIY---

ZmPSTOL8.05_1 LKDT-QGDGEEFMNEVASISRTSHVNVVTLLGFCLQGSKRALIYEYMPNGSLERYAF-TG

ZmPSTOL8.05_2 LKDT-QGDGEEFMNEVASISRTSHVNVVTLLGFCLQGSKRALIYEYMPNGSLERYAF-TG

* . :******:*** .*.:*.*:*:*.* *:.*:*.****:****.****::* :

OsPSTOL1 SVQGDNTLSWDRLFNIIVGIARGLEYLHCHCNIRIVHFDIKPQNILLAQDFCPKISDFGL

ZmPSTOL3.04 SDNPKEVLGWDRLYTIAIGIARGLEYLHHSCNTRIVHFDIKPQNILLDQNFQPKIADFGL

ZmPSTOL3.06 --RAENTLSWEKLFDVATGTARGLEYLHRGCNTPIVHFDIKPHNILLDQDFCPKISDFGL

ZmPSTOL4.05 SSSGEDALSWDRLFGIVVGVARGLEYLHTGCNTRIVHFDIKPHNILLDQDMCPKISDFGL

ZmPSTOL8.02 TENPKAVLGWDKLYTIAIGIARGLEYLHHSCNTRIVHFDIKPQNILLDQNFHPKIADFGL

ZmPSTOL8.05_1 DMNSENLLTWERLFDIAIGTARGLEYLHRGCNTRIVHFDIKPHNILLDQDFCPKISDFGL

ZmPSTOL8.05_2 DMNSENLLSWERLFDIAIGTARGLEYLHRGCNTRIVHFDIKPHNILLDQDFCPKISDFGL

. * *::*: : * ******** ** ********:**** *:: ***:****

OsPSTOL1 SKLCHLK-ESRISINGLRGTPGYIAPEVFSRQYGSASSKSDVYSYGMVVLEMAGAK-KNI

ZmPSTOL3.04 AKLCHTK-ESKLSMTGARGTPGFIAPEVHSRTFGVVSTKSDVYSYGMMLLEMVGGRKNVK

ZmPSTOL3.06 AKLCPNKASSAVSIVGARGTVGYIAPEVYSKQFGVVSSKSDVYSYGMMVLEMVGARDKST

ZmPSTOL4.05 AKLCGQK-ASRVSIAGARGTVGYIAPEVFSRSYEAVGSKADVYSYGMVVLEMVGAR-KNV

ZmPSTOL8.02 AKLCHMK-ESKLSMTGARGTPGFIAPEVHSRTFGVVSTKADVYSYGMMLLEMVGGRKNVN

ZmPSTOL8.05_1 AKLCLNK-ESAISIVGARGTIGYIAPEVYSKQFGTISSKSDVYSYGMMVLEMVGARDRNT

ZmPSTOL8.05_2 AKLCLNK-ESAISIVGARGTIGYIAPEVYSKQFGTISSKSDVYSYGMMVLEMVGARDRNT

:*** * * :*: * *** *:*****.*: : .:*:*******::***.*.: .

OsPSTOL1 NVS------TGSSSKYFPQWLYDNL-DQFCCPTGEISSQ-------TTD--LVRKMVVVG

ZmPSTOL3.04 SFA------QVSSEKYFPHWIYDHFGQNDGLLACEGTPE-------KEE--IAKKMALVG

ZmPSTOL3.06 SAD------SERSSQYFPQWIYEHL-DDYCVSASEVDGG-------TTE--LVRKMIVVG

ZmPSTOL4.05 HVSATDDGGNSSSSRYFPQWLYENL-DQFCRPTTTSNGEIRGDDDDATEVLLVRKMVVVG

ZmPSTOL8.02 SAA------QESSEKYFPHWIYDHFGQEDGLQACEVTRE-------NEG--IAKKLSVIG

ZmPSTOL8.05_1 SAD------SDHSSQYFPQWLYEHL-DDYCVGASEINGE-------TTE--LVRKMIVVG

ZmPSTOL8.05_2 SAD------SDHSSQYFPQWLYEHL-DDYCVGASEINGE-------TTE--LVRKMIVVG

*.:***:*:*::: :: : :.:*: ::*

OsPSTOL1 LWCIQLVPTDRPSMREVLEMLESNGRDLPLPPKGL-------------------------

ZmPSTOL3.04 MWCIQILPLHRPTITKVLEMFDRGLDELDMPPRQNFSQTFEDPTYNFNAENMSTSSGTKT

ZmPSTOL3.06 MWCIQLIPTDRPTMTRVVEMLEGSTSNLELPPKVLLSWQASIDVFLLRPQLI--------

ZmPSTOL4.05 LWCIQSKPDSRPSMGQVLEMLESNAADLQLPPKALCT-----------------------

ZmPSTOL8.02 LSCIQILPMHRPTIGKVMEMFERGSDELDMPPRQNFSQIFEDPVHSLNAETMSMISGTKA

ZmPSTOL8.05_1 LWCIQVIPTDRPTMTRVVEMLEGSTSNLELPPRVLLS-----------------------

ZmPSTOL8.05_2 LWCIQVIPTDRPTMTRVVEMLEGSTSNLELPPRVLLS-----------------------

: *** * **:: .*:**:: . :* :**:

OsPSTOL1 --------------------------

ZmPSTOL3.04 QR------------------------

ZmPSTOL3.06 KGY-----------IN----------

ZmPSTOL4.05 -AY-----------------------

ZmPSTOL8.02 KAYSEVLKMKEISVVNSKTIQRLPTL

ZmPSTOL8.05_1 --------------------------

ZmPSTOL8.05_2 --------------------------

* = identical amino acids residues in all sequences

: = highly conserved amino acids

. = different but somewhat similar amino acid

blank = dissimilar amino acids or gaps
