# Supplementary material for: Multiple interval QTL mapping and searching for PSTOL1 homologs associated with root morphology, biomass accumulation and phosphorus content in maize seedlings under low-P
Source: BMC Plant Biol. 2015 Jul 7;15:172. doi: 10.1186/s12870-015-0561-y (PMC4492167; doi:10.1186/s12870-015-0561-y)
Supplement: Additional file 4: Table S2. — Total P content in the seedling and relative expression of ZmPSTOL genes in shoots and roots of both parental lines in low- and high-P concentration. [file 12870_2015_561_MOESM4_ESM.docx]

**Table S2** Total P content in the seedling and relative expression of *ZmPSTOL* genes in shoots and roots of both parental lines in low- and high-P concentration

|  | **Total P Content (mg)** | | |  | | | **Relative Gene Expression (RQ)** | | | | | | | | | | | | | | | | | | | | | | | | | | | | | | | | | | | | | | | | | | | | | | | | | | | | | | | | | | | | | | | | | | | | | | | | | |  | |  |
| --- | --- | --- | --- | --- | --- | --- | --- | --- | --- | --- | --- | --- | --- | --- | --- | --- | --- | --- | --- | --- | --- | --- | --- | --- | --- | --- | --- | --- | --- | --- | --- | --- | --- | --- | --- | --- | --- | --- | --- | --- | --- | --- | --- | --- | --- | --- | --- | --- | --- | --- | --- | --- | --- | --- | --- | --- | --- | --- | --- | --- | --- | --- | --- | --- | --- | --- | --- | --- | --- | --- | --- | --- | --- | --- | --- | --- | --- | --- | --- | --- | --- | --- | --- |
|  |  |  |  |  | | | *ZmPSTOL3.04* | | | | | | | |  | | *ZmPSTOL3.06* | | | | | | |  | |  | | *ZmPSTOL4.05* | | | | | | | | |  | | |  | | | *ZmPSTOL8.02* | | | | | | | |  | |  | | | | *ZmPSTOL8.05_1* | | | | | | | | | |  | | |  | | | *ZmPSTOL8.05_2* | | | | | | | |  |  |  |
|  |  |  |  |  | | | Root | | |  | | | Shoot | |  | | Root | |  | | | Shoot | |  | |  | | Root | | |  | | | Shoot | | |  | | |  | | | Root | | |  | Shoot | | | | |  | | |  | | | | Root | |  | | Shoot | | |  | | |  | | | Root | | |  | | Shoot | |  | | | | |
| L3 low P | | 0.644 a |  | | 1.01 | | | b | | | 1.27 b | | |  | | 1.01 | | a | | | 0.12 | | a | |  | | 1.05 | | | b | | | 0.86 | | a | | |  | | | 1.05 | | | b | | | | 0.79 | | c | | | | | |  | | | | 1.03 | | b | | 1.54 | a | | |  | | | 1.09 | | | c | | 0.80 | | c | |  |  |  |  |
| L3 high P | | 0.687 a |  | | 0.86 | | | b | | | 0.84 c | | |  | | 0.75 | | b | | | 0.11 | | a | |  | | 1.05 | | | b | | | 0.77 | | a | | |  | | | 0.68 | | | b | | | | 1.13 | | b | | | | | |  | | | | 1.51 | | b | | 2.66 | a | | |  | | | 0.35 | | | c | | 0.34 | | c | |  |  |  |  |
| L22 low P | | 0.515 a |  | | 0.32 | | | c | | | 2.29 a | | |  | | 0.21 | | d | | | 0.02 | | b | |  | | 3.28 | | | a | | | 0.15 | | a | | |  | | | 5.59 | | | a | | | | 1.22 | | a | | | | | |  | | | | 23.48 | | a | | 2.11 | a | | |  | | | 3.19 | | | b | | 11.18 | | a | |  |  |  |  |
| L22 high P | | 0.637 a |  | | 1.87 | | | a | | | 0.27 d | | |  | | 0.50 | | c | | | 0.01 | | b | |  | | 2.66 | | | a | | | 0.24 | | a | | |  | | | 7.33 | | | a | | | | 0.64 | | d | | | | | |  | | | | 22.75 | | a | | 3.96 | a | | |  | | | 7.36 | | | a | | 3.05 | | b | |  |  |  |  |
| LS mean | | 0.131 |  | | | 0.25 | | |  | | | 0.33 | |  | | 0.20 | |  | | 0.03 | | |  | |  | | 1.08 | |  | | | 1.88 | | | |  | | |  | | | 2.02 | | |  | | | | 0.08 |  | | | |  | | | | 8.07 | | | |  | | 5.31 |  | | |  | | | 1.06 | | |  | | 0.74 | |  | |  | |  |  |

Different letters following the mean values indicate statistical differences by t-test at 5% of probability
