# Supplementary material for: Multiple interval QTL mapping and searching for PSTOL1 homologs associated with root morphology, biomass accumulation and phosphorus content in maize seedlings under low-P
Source: BMC Plant Biol. 2015 Jul 7;15:172. doi: 10.1186/s12870-015-0561-y (PMC4492167; doi:10.1186/s12870-015-0561-y)
Supplement: Additional file 5: Table S3. — Primers used for gene expression analyses. [file 12870_2015_561_MOESM5_ESM.docx]

**Additional File 5: Table S3 Primers used for gene expression analyses**

| **Gene ID** | **Primer** | **Chemical detection** | **Primer sequence (5' - 3')** | **Probe sequence** | **Product length (bp)** |
| --- | --- | --- | --- | --- | --- |
| *ZmPSTOL3.04* | **Zm3.04_1F** | TaqMan | ACCATAGCAATTGGGATCGC | TCGAGGGTTGGAATACTTGCACCAT | 75 |
|  | **Zm3.04_1R** |  | ATCGAAATGGACTATGCGTGT |  |  |
|  | Zm3.04_2F | TaqMan | CCTGGGTTTATAGCTCCTGAAG | TGTTACTAGAGATGGTTGGAGGCAGGA | 141 |
|  | Zm3.04_2R |  | ACTTGAAACTTGAGCAAATGATTTAAC |  |  |
| *ZmPSTOL3.06* | **Zm3.06_1F** | TaqMan | TCAACATCGTGACACTGACG | CATGTACTCGTAAACAAGCGCCCTCT | 92 |
|  | **Zm3.06_1R** |  | GTACCTTTCAAGCGAACCATTC |  |  |
|  | Zm3.06_2F | TaqMan | AGTATCAGCAGGACTTGTCATG | CAAGCAGAACCCCGTCAGTGTCA | 97 |
|  | Zm3.06_2R |  | CGCCCTCTTGGATCCTTG |  |  |
| *ZmPSTOL4.05* | **Zm4.05_1F** | SYBR Green | GTTACAGTGCCCGGATTCTGA |  | 110 |
|  | **Zm4.05_1R** |  | ACGTCTCTTGTACCACCACATCA |  |  |
| *ZmPSTOL8.02* | **Zm8.02_1F** | TaqMan | TGGTTTTCAAGGGAAGGCTAG | CAGGAATTTCACTGCAACTAGACGACCA | 73 |
|  | **Zm8.02_1R** |  | CCGTCACCTTTGGAGTCATG |  |  |
|  | Zm8.02_2F | SYBR Green | CCAGTACACAGTTTGAATGCG |  | 133 |
|  | Zm8.02_2R |  | GTTTTACAGAGTTGGCAACCG |  |  |
| *ZmPSTOL8.05-1* | Zm8.05.1_1F | TaqMan | ATCAAAAAGAAAAGAAGCAGCA | AACGGCAACAGCACCAACAATAGG | 78 |
|  | Zm8.05.1_1R |  | AAGGATGTGAGAATGACTAGACAC |  |  |
|  | **Zm8.05.1_2F** | SYBR Green | AGAAGCAGCACCTATTGTTGG |  | 73 |
|  | **Zm8.05.1_2R** |  | AGCCAAGAAGGATGTGAGAATG |  |  |
| *ZmPSTOL8.05-2* | **Zm8.05.2_1F** | TaqMan | GGGCATACGGACGTACC | CTTGAATTCCGGCTTGGGAAGTTGG | 74 |
|  | **Zm8.05.2_1R** |  | ACCGTCTTCATATGGTCAACC |  |  |
|  | Zm8.05.2_2F | TaqMan | TGGAGGTATCTGGAAAGAAGC | CGTATGCCCCTCCTCCACCTG | 84 |
|  | Zm8.05.2_2R |  | ATTCAAGCCTCATTCCCGG |  |  |
| 18S | 18S_Zm_F | SYBR Green | CGTCCTAGTCTCAACCATAAACG |  | 82 |
|  | 18S_Zm_R |  | CCCCGGAACCCAAAGACT |  |  |

Results with primers highlighted in bold are presented on Figure 4.

18S is a primer pair re-designed from the TaqMan^®^ Eukaryotic 18S rRNA (Applied Biosystems, Foster City, CA) and used as endogenous control.
